# Association between AT1 receptor gene polymorphism and left ventricular hypertrophy and arterial stiffness in essential hypertension patients: a prospective cohort study

**DOI:** 10.1186/s12872-022-03024-7

**Published:** 2022-12-28

**Authors:** Hangjun Ou, Danan Liu, Guangjian Zhao, Caiwei Gong, Yunyun Li, Quanwei Zhao

**Affiliations:** Department of Cardiology, Affiliated Hospital of Guizhou Medical University, Guiyang, 550004 Guizhou China

**Keywords:** AGTR1, Genetic polymorphism, Hypertension, Left ventricular hypertrophy, Arterial stiffness, Essential hypertension, Cohort study

## Abstract

**Background:**

AT1 receptor gene (*AGTR1*) is related to essential hypertension (EH), and left ventricular hypertrophy (LVH) and arterial stiffness are common complications of EH. This study aimed to explore the association between *AGTR1* genotype and LVH and arterial stiffness in EH patients.

**Methods:**

A total of 179 EH patients were recruited in this study. Oral exfoliated cells were collected from each patient, and the genetic polymorphism of *AGTR1*(rs4524238) was assessed using a gene sequencing platform. The outcomes were LVH and arterial stiffness.

**Results:**

Among 179 patients, 114 were with *AGTR1* genotype of GG (57 males, aged 59.54 ± 13.49 years) and 65 were with AGTR1 genotype of GA or AA (36 males, aged 61.28 ± 12.79 years). Patients with AGTR1 genotype of GG were more likely to have LVH (47 [41.23%] vs. 14 [21.54%], *P* = 0.006) and arterial stiffness (30 [26.32%] vs. 8 [12.31%], *P* = 0.036). The AGTR1 polymorphism frequency was in accordance with Hardy–Weinberg equilibrium (*P* = 0.291). The multivariate logistic regression showed that AGTR1 genotype of GA or AA was independently associated with lower risk of LVH (OR = 0.344, 95%CI 160~0.696, *P* = 0.003) and arterial stiffness (OR = 0.371, 95%CI 0.155~0.885, *P* = 0.025) after adjusting for gender, age, and diabetes.

**Conclusion:**

EH patients with the AGTR1 genotype of GA or AA were at lower risk for LVH and arterial stiffness than those with the GG genotype.

## Background

Hypertension is one of the most common chronic diseases in China. According to the China Hypertension Survey, the number of adults with hypertension is approximately 245 million [1]. Therefore, China’s potential health burden of future cardiovascular events remains high. Essential hypertension (EH) is a common form of hypertension that accounts for 95% of all hypertension cases. EH is an inherited disease caused by multiple genetic and environmental factors [2, 3]. Several potential candidate genes and genomic regions are associated with the pathogenesis of blood pressure variability and target organ damage. Also, polymorphisms in some of these candidate genes have been reported to be associated with the progression of hypertension, where polymorphisms in the renin-angiotensin system (RAS) are undoubtedly the most prominent of these candidates [4–6].

Prolonged poor blood pressure control in patients with hypertension may cause damage to target organs such as blood vessels, heart, brain, and kidneys. Left ventricular hypertrophy (LVH) is a manifestation caused by prolonged increased cardiac workload and is most commonly associated with hypertension. Studies have shown that the prevalence of hypertension combined with LVH is as high as 57.5% [7]. Hypertension is usually associated with more severe nocturnal hypoxemia, elevated systolic blood pressure, and increased body mass index (BMI). In addition, hypertension increases intravascular luminal pressure and disrupts the vascular media elastin, producing and depositing large amounts of collagen in the media, resulting in impaired arterial elasticity and arterial stiffness [8]. Arterial stiffness represents the stiffness of the arterial wall. Arterial stiffness often increases with abnormal changes in vessel wall composition and damage to the media. Typical risk factors for cardiovascular diseases, such as high blood pressure and cholesterol, stress, smoking, and obesity, have a negative impact on arterial stiffness [9]. Among them, chronic hypertension amplifies changes in the vessel wall and increases vessel stiffness; increased aortic stiffness feeds back into blood pressure and increases systolic blood pressure. Therefore, the assessment of arterial stiffness is important for preventing and treating hypertension and its complications.

Considering the role of genetic susceptibility in the development and progression of hypertension, several research strategies have been used for early detection in high-risk populations and the development of new therapies for the prevention or treatment of hypertension [9]. Molecular epidemiological studies of single nucleotide polymorphisms (SNPs) of candidate genes have become an effective way to explore the pathogenesis of hypertension [10]. To date, more than 150 candidate genes for hypertension have been identified, and the human AT1 receptor gene (AGTR1) is one of them. Many studies have also shown that *AGTR1* gene polymorphisms are closely associated with the development of arterial stiffness by acting in combination with other gene polymorphisms or alone [11].

*AGTR1* is commonly expressed in the human organism, which locates on chromosome 3q21-25 and encodes the type 1 angiotensin II receptor (AGTR1)[12]. Related investigations have shown that *AGTR1* polymorphisms are associated with blood pressure responses to RAS inhibition in hypertensive populations. E.g., AGTR1(rs5186) affects the antihypertensive function of angiotensin II receptor antagonists by altering the sensitivity of these receptors. Also, variants in AGTR1 (rs4524238 and rs3772616) have close relationship with blood pressure responses to low-sodium intervention, and individual differences in blood pressure lowering by candesartan are associated with AGTR1 polymorphisms and their genotypic expression [13–17].

While existing studies suggested that the AGTR1 polymorphism is closely associated with hypertension, it remains unclear whether its genotype is associated with LVH and arterial stiffness. Therefore, the aim of this study was to explore the association between AGTR1 polymorphism (rs4524238) and LVH and arterial stiffness in EH patients.

## Methods

### Study design and patients

This prospective cohort study included EH patients treated in the Department of Cardiovascular Medicine at the Affiliated Hospital of Guizhou Medical University from October 2018 to January 2020. The inclusion criteria were the following: (1) age ≥ 18 years; (2) blood pressure (BP) was measured at the time of visit more than three times without antihypertensive treatment; (3) systolic blood pressure (SBP) > 140 mmHg and diastolic blood pressure (DBP) > 90 mmHg. Exclusion criteria were: patients with secondary hypertension or renal disease and endocrine disease.

The study was approved by the Ethics Committee of the Affiliated Hospital of Guizhou Medical University [R19027]. All enrolled patients understood the study content and signed the written informed consent.

### Procedure

#### Genotyping of AGTR1 (rs4524238)

Oral exfoliated cells from all patients were collected and placed in collection tubes. DNA extraction was performed using the TIANamp Blood DNA Kit (TIANGEN Biotechnology, Beijing, China) according to the manufacturer’s instructions, and the obtained genomes were accurately quantified using the Qubit® dsDNA HS Assay Kit. Polymerase chain reaction (PCR) (Table 1) was followed by sequencing of the DNA products using the Illumina X10 high-throughput sequencing platform, after which bioinformatics analysis of the raw data was obtained by sequencing and generation of reports.

**Table 1:** Polymerase chain reaction process

Protocol	Temperature (°C)	Time (s)	
Pre-denaturation	95	30	30 cycles
Denaturation	95	10	
Annealing	60	30	
Extension	70	30	

#### Cf-PWV determination

Carotid-femoral pulse wave velocity (cf-PWV) was measured using the CompliorsP arterial pulse wave velocity detector. All patients were asked to refrain from smoking, drinking, and eating for 3 h before the examination. The examination room was quiet with comfortable temperature. Patients were instructed to rest in the supine position in the examination room for 15 min. The vertical distance from the carotid artery to the body surface of the femoral artery was measured with a tape measure and entered into the computer. The pressure transducer was then placed on the right carotid and femoral arteries with the most pronounced pulsation, and the instrument automatically detected the cf-PWV value. The above operations were performed by qualified personnel with uniform training. Higher cf-PWV values indicated higher arterial stiffness. According to the 2018 ESC/ESH Guidelines for the management of arterial hypertension and the Task Force for the management of arterial hypertension of the European Society of Cardiology (ESC) and the European Society of Hypertension (ESH), all participants were divided into 2 classes: class 1 (cf-PWV > 10 m/s); class 2 (cf-PWV ≤ 10 m/s).

#### LVMI determination

Left ventricular end-diastolic dimension (LVDd), septal thickness (LVST), and left ventricular posterior wall thickness (LVPWT) were measured by the same technician using color Doppler ultrasound. Left ventricular mass (LVM) (g) = 0.8 × 1.04 × [(LVST + LVPWT + LVDd)^3^- LVDd^3^] + 0.6, body surface area (BSA) = 0.0061 × height + 0.0128 × weight − 0.1529, and left ventricular mass index (LVMI) (g/m^2^) = LVM/BSA calculated according to the Devereux correction formula. The diagnostic criteria for LVH were LVMI > 115 g/m^2^ (men) and > 95 g/m^2^ (women) [18].

### Outcome and data collection

The outcomes in this study were LVH and arterial stiffness (cf-PWV). The demographic information (including age, sex, BMI, smoking, and alcohol consumption), clinical characteristics (including SBP, DBP, central arterial systolic blood pressure [cSBP], triglyceride [TG], total cholesterol [TC], high-density lipoprotein cholesterol [HDL-C], low-density lipoprotein cholesterol [LDL-C], urinary sodium, and serum creatinine [Scr], genotype, and allele of AGTR1 gene were collected.

### Statistical analysis

Statistical Package for Social Sciences 22.0 (IBM, Armonk, New York, USA) was used for data analysis. The continuous data were expressed as mean ± standard deviation (SD) and compared by t-test. Categorical data were expressed as n (%) and compared by the chi-square test. Hardy-Weinberg equilibrium was tested by the fit chi-square test. Multivariate logistic regression analysis was used to explore the association between AGTR1 genotype with LVH and arterial stiffness after adjusting for gender, age, and diabetes (Insignificant factors had been omitted after preliminary statistics). A two-sided P < 0.05 was considered statistically significant.

## Results

A total of 179 patients were finally included in this study. Among them, 114 were with AGTR1 genotype of GG (57 males, aged 59.54 ± 13.49 years), and 65 patients were with of GA or AA genotype (36 males, aged 61.28 ± 12.79 years).

Patients with GG genotype were more likely to have lower BMI (23.71 ± 3.13 vs. 25.16 ± 3.24, *P* = 0.008), higher SBP (146.52 ± 24.74 vs. 138.15 ± 21.06, *P* = 0.027), higher Csbp (143.07 ± 22.87 vs. 133.60 ± 20.30, *P* = 0.006), higher TG (**2.22** ± 1.86 vs. **1.61** ± 1.05, *P* = 0.032), and higher Scr (**91.52** ± 46.29 vs. 69.80 ± 14.56, *P* = 0.017) compared to GA or AA genotype. Patients with GG genotype were more likely to have LVH (47 [41.23%] vs. 14 [21.54%], *P* = 0.006) and arterial stiffness (30 [26.32%] vs. 8 [12.31%], *P* = 0.036) (Table 2). The genotype distribution of AGTR1 in patients was in accordance with the Hardy–Weinberg equilibrium (*P* = 0.291) (Table 3).

**Table 2:** Baseline characteristics of patients

Characteristics	GG (n = 114)	GA + AA (n = 65)	P
Age, years	59.54 ± 13.49	61.28 ± 12.79	0.401
Gender			0.535
Male	57 (50.00)	36 (55.38)	
Female	57 (50.00)	29 (44.62)	
BMI, Kg/m^2^	23.71 ± 3.13	25.16 ± 3.24	0.008
Smoker	23 (20.18)	15 (23.08)	0.705
Drinker	22 (19.30)	13 (20.00)	1.000
Diabetic	14 (12.28)	14 (21.54)	0.134
SBP (mmHg)	146.52 ± 24.74	138.15 ± 21.06	0.027
DBP (mmHg)	79.76 ± 20.63	83.77 ± 13.22	0.135
Csbp (mmHg)	143.07 ± 22.87	133.60 ± 20.30	0.006
TG (mmol/l)	2.22 ± 1.86	1.61 ± 1.05	0.032
TC (mmol/l)	4.00 ± 1.09	4.09 ± 1.00	0.585
HDL-C (mmol/l)	1.26 ± 0.60	1.16 ± 0.33	0.156
LDL-C (mmol/l)	2.82 ± 0.91	2.80 ± 0.79	0.924
Urinary sodium/24 h (mmol/l)	142.24 ± 63.83	131.14 ± 56.30	0.289
Scr (µmol/l)	91.52 ± 46.29	69.80 ± 14.56	0.017
LVH			0.006
LVH	47 (41.23)	14 (21.54)	
NLVH	67 (58.77)	51 (78.46)	
Grades of cf-PWV			0.036
Grade 1	30 (26.32)	8 (12.31)	
Grade 2	84 (73.68)	57 (87.69)	

Data were expressed as mean ± standard deviation (SD), and n (%)

*SBP* systolic blood pressure, *DBP* diastolic blood pressure, *cSBP* central arterial systolic blood pressure, *TG* triglyceride, *TC* total cholesterol, *HDL-C* high density lipoprotein cholesterol, *LDL-C* low density lipoprotein cholesterol, *LVH* left ventricular hypertrophy, *NLVH* non-left ventricular hypertrophy, *cf-PWV* carotid-femoral pulse wave velocity

**Table 3:** The genotype distribution of AGTR1 and Hardy-Weinberg equilibrium in patients

Genotype	Observed number (N)	Expected number (N)	Frequency (%)	P
AGTR1-GG	114	118	63.7	0.291
AGTR1-GA	63	55	35.2	
AGTR1-AA	2	6	1.1	

**Fig. 1:**
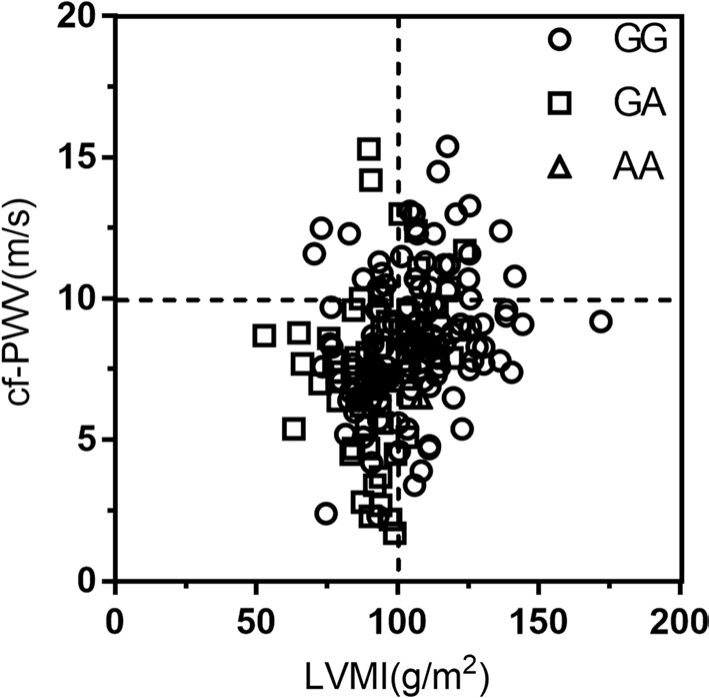
The genotype distribution of AGTR1 on the cf-PWV- LVMI axis

The multivariate logistic regression analysis showed that AGTR1 genotype of GA or AA was independently associated with lower risk of LVH (OR = 0.344, 95% CI 0.160 ~ 0.696, *P* = 0.003) and arterial stiffness (OR = 0.371, 95% CI 0.155 ~ 0.885, *P* = 0.025) after adjusting gender, age, and diabetes (Table 4). The distribution of arterial stiffness combined with LVH in different AGTR1 genotypes supported that the distribution of arterial stiffness and LVH was significantly different among genotypes, and patients with AGTR1 GG genotype were more likely to have both complications, which is also consistent with the above results (Fig. 1).

**Table 4:** Genotype, LVH, and arterial stiffness in patients

Characteristics	OR	95%CI	*P*
LVH			
Genotype (GG vs. GA + AA)	0.334	0.160 ~ 0.696	0.003
Cf-PWV Grade			
Genotype (GG vs. GA + AA)	0.371	0.155 ~ 0.885	0.025

Adjusted with gender, age, and diabetes. *LVH* left ventricular hypertrophy,* cf-PWV* carotid-femoral pulse wave velocity

## Discussion

The present study found that EH patients with the AGTR1 (rs4524238) genotype of GA or AA might be at lower risk for LVH and arterial stiffness compared to the GG genotype. And G allele of AGTR1 appears to be more strongly associated with SBP, which is consistent with the study of Felder et al. [19]. On the contrary, one research has indicated that the A allele has closer relationship with increased DBP and mean arterial pressure (MAP) [20]. These inconsistent conclusions may be due to different samples sizes.

Long-term poor blood pressure control may lead to target organ damage and increased cardiac load, which in turn leads to LVH. LVH is not only an adaptive response to hemodynamic changes in hypertension patients but also a risk factor for adverse cardiovascular events. This study showed that the GG genotype of the AGTR1 gene was the most common genotype in patients with hypertension and LVH, and the distribution of AGTR1 polymorphisms was significantly different in the LVH and non-LVH patients. A previous study showed that LVMI was associated with the AGTR1 A1166C polymorphism, which might be mediated by different expressions of AGTR1 as modulated by microRNA-155 [21]. Besides, it was reported that LVMI might be greater in the presence of ACE- DD and AGTR1-AC/CC polymorphisms in endurance athletes [22, 23], which indicates that AGTR-1 polymorphisms promote LVH not only in patients with hypertension but also in healthy individuals with elevated LVMI. These aforementioned studies were consistent with the present study. However, some studies found no significant association between AGTR1 polymorphisms and LVH [24, 25]. The effect of AGTR1 gene polymorphism on LVH may vary among different ethnic groups and populations, so further studies are needed to confirm this conjecture.

The assessment of arterial stiffness is important for preventing and treating hypertension and developing complications. The volume of blood injected into the aorta during systole generates a wave (pulse wave) that circulates through the arterial system at a certain velocity. Cf-PWV measurement has now become the “gold standard” for noninvasive assessment of arterial stiffness, where cf-PWV > 10 m/s is considered a conservative estimate of changes in aortic function in middle-aged hypertension patients. This study showed an association between AGTR1 and arterial stiffness, and patients with the AGTR1 genotype of GG were more likely to suffer from arterial stiffness. Marcin et al. reported that the AGTR1 genotype was not related to vascular stiffness; however, other studies confirmed that AGTR1 gene polymorphism is closely associated with the occurrence of arterial stiffness by combining with other gene polymorphisms or acting alone [26, 27]. This indicates that AGTR1 gene polymorphism is involved in the arterial stiffness of hypertension patients, which is consistent with the present study.

There are several limitations to this study. First, due to the small sample of the study population, with only 2 patients with the AA genotype, additional sample size is needed in this study to clarify the relationship between different AGTR1 genotypes and their complications. Second, this study only included EH patients, so reported results could not be generalized to all patients with hypertension.

In conclusion, EH patients with the AGTR1 genotype of GA or AA might be at lower risk for LVH and arterial stiffness compared to the GG genotype. EH patients with AGTR1 genotype of GG need examination for LVH and arterial stiffness to receive appropriate treatment.

## Data Availability

The datasets used and/or analyzed during the current study are available from the corresponding author upon reasonable request.

## Abbreviations

GTR1: AT1 receptor gene
EH: Essential hypertension
RAS: Renin-angiotensin system
LVH: Left ventricular hypertension
BMI: Body mass index
PCR: Polymerase chain reaction
LVH: Left ventricular hypertrophy
PCR: Polymerase chain reaction
ESC: European society of cardiology
